# An *Arabidopsis* Nucleoporin *NUP85* modulates plant responses to ABA and salt stress

**DOI:** 10.1371/journal.pgen.1007124

**Published:** 2017-12-12

**Authors:** Yingfang Zhu, Bangshing Wang, Kai Tang, Chuan-Chih Hsu, Shaojun Xie, Hai Du, Yuting Yang, Weiguo Andy Tao, Jian-Kang Zhu

**Affiliations:** 1 Shanghai Center for Plant Stress Biology and CAS Center of Excellence for Molecular Plant Sciences, Chinese Academy of Sciences, Shanghai, China; 2 Department of Horticulture and Landscape Architecture, Purdue University, West Lafayette, IN, United States of America; 3 Department of Biochemistry, Purdue University, West Lafayette, IN, United States of America; 4 College of Agronomy and Biotechnology, Southwest University, Chongqing, China; 5 Key Laboratory of Sugarcane Biology and Genetic Breeding Ministry of Agriculture, Fujian Agriculture and Forestry University Fuzhou, Fuzhou, China; Duke University, UNITED STATES

## Abstract

Several nucleoporins in the nuclear pore complex (NPC) have been reported to be involved in abiotic stress responses in plants. However, the molecular mechanism of how NPC regulates abiotic stress responses, especially the expression of stress responsive genes remains poorly understood. From a forward genetics screen using an abiotic stress-responsive luciferase reporter (*RD29A-LUC*) in the *sickle-1* (*sic-1*) mutant background, we identified a suppressor caused by a mutation in *NUCLEOPORIN 85* (*NUP85*), which exhibited reduced expression of *RD29A-LUC* in response to ABA and salt stress. Consistently, the ABA and salinity induced expression of several stress responsive genes such as *RD29A*, *COR15A* and *COR47* was significantly compromised in *nup85* mutants and other nucleoporin mutants such as *nup160* and *hos1*. Subsequently, Immunoprecipitation and mass spectrometry analysis revealed that NUP85 is potentially associated with HOS1 and other nucleoporins within the nup107-160 complex, along with several mediator subunits. We further showed that there is a direct physical interaction between MED18 and NUP85. Similar to *NUP85* mutations, *MED18* mutation was also found to attenuate expression of stress responsive genes. Taken together, we not only revealed the involvement of *NUP85* and other nucleoporins in regulating ABA and salt stress responses, but also uncovered a potential relation between NPC and mediator complex in modulating the gene expression in plants.

## Author summary

Nuclear pore complex (NPC) mediates the traffic between nucleus and cytoplasm. This work identified *NUCLEOPORIN 85* (*NUP85*) as an important factor for the expression of stress-responsive luciferase reporter gene *RD29A-LUC* in response to ABA and salt stress from a forward genetics screen. Mutation in *NUP85* and other NPC components such as *NUP160* and *HOS1* resulted in decreased expression of several stress responsive genes such as *RD29A*, *COR15A* and *COR47*. Proteomics data uncovered a list of putative NUP85 associated proteins. Furthermore, NUP85 was demonstrated to interact with MED18, a master transcriptional regulator, to control the expression of stress responsive genes. The study has added a new layer of knowledge about the diverse functions of NPC in abiotic stress responses.

## Introduction

Nucleocytoplasmic transport plays vital roles in eukaryotic systems [1,2]. The exchange of macromolecules such as RNAs and proteins is predominantly regulated by highly conserved nuclear pore complexes (NPCs), which consist of multi-nucleoporins (Nups) arranged in distinct sub-complexes [3,4]. Although the composition and functions of NPCs have been extensively characterized in yeast and vertebrate systems, our knowledge on the functions of plant NPCs remains poor.

Recent genetic screens have identified the involvement of several Nups from *Arabidopsis* and *Lotus japonicus* (lotus) in a variety of developmental processes, hormone signaling pathways and environment adaptation [3,5,6]. For instance, *NUP160* (*SUPPRESSOR OF AUXIN RESISTANCE1*) and *NUP96* (*SUPPRESSOR OF AUXIN RESISTANCE3*) have been indicated to play a role in auxin signaling as they were identified as suppressors of *auxin-resistant 1*(*axr1*) mutants [7]. *NUP160* was also shown to be involved in cold stress responses since the knock-out of *NUP160* impaired cold-induced expression the *CBF3-LUC* reporter gene and several endogenous genes; and thus resulted in hypersensitivity to chilling and freezing stresses [8]. Another important NPC component [9], *HIGH EXPRESSION OF OSMOTICALLY RESPONSIVE GENES1* (*HOS1*), which encodes a RING finger E3 ubiquitin ligase, is well-known as a negative regulator in the cold signaling [10]. Cold-responsive genes such as *RESPONSIVE TO DESICCATION 29A* (*RD29A*), *COLD-REGULATED 47* (*COR47*), *COLD-REGULATED 15A* (*COR15A*) and *KIN1* were reported to be induced to higher levels in *hos1* mutants than in wild type plants [11,12]. HOS1 was further shown to attenuate cold signaling by ubiquitination and degradation of INDUCER OF CBF EXPRESSION 1 (ICE1), which encodes an important positive transcription factor critical for the cold-induction of *C-REPEAT BINDING FACTORs* (*CBFs*) [11–13]. Moreover, the mutation of *NUP160*, *HOS1* and *NUP96* caused early flowering phenotypes, indicating that NPCs are also involved in flowering time regulation [5]. Interestingly, only *HOS1* was demonstrated to interact with specific chromatin such as the floral repressor *FLOWERING LOCUS C* (*FLC*) chromatin to regulate *FLC* transcription under low temperature [14–16].

Besides their involvement in hormonal and cold stress signaling and flowering time control, some Nups such as *NUP160* and *Seh1* are also important for disease resistance in *Arabidopsis* [17,18]. Similarly, mutation of either of *NUP85*, *Seh1* and *NUP133* led to defective responses to symbiotic microorganisms in *Lotus Japonicus* [19,20]. Most recent genetic studies have revealed *NUP85* (also termed as *SBB1*, *s**uppressor of* *b**ak1* *b**kk1-1*) as a suppressor for BRI1-ASSOCIATED KINASE 1 (BAK1) and BAK1-LIKE 1 (BKK1)-mediated cell death control [21]. These studies suggested that Nups also participate in plant defense and cell death control. Nevertheless, the biological functions of many Nups remain elusive in plants.

Recently, a proline-rich protein gene, *SICKLE-1* (*SIC-1*), was isolated because *sic-1* mutants exhibited enhanced expression of stress-inducible *RD29A-LUC* reporter in response to abiotic stresses such as cold and salt treatments [22]. To identify new regulatory components involved in the response to abiotic stresses, we performed a forward genetic screen after EMS mutagenesis of the *RD29Apro-LUC* line in the *sic-1* mutant background. *NUP85* was identified as its mutation caused significantly reduced expression of *RD29A-LUC* in response to ABA and salt stress. Moreover, we discovered a list of putative NUP85 interacting proteins by affinity purification followed by mass spectrometry and further validated the interaction between NUP85 and MED18.

## Results

### The *nup85* mutation attenuates *proRD29A-LUC* reporter gene expression in response to ABA and salt stress

**Fig 1:**
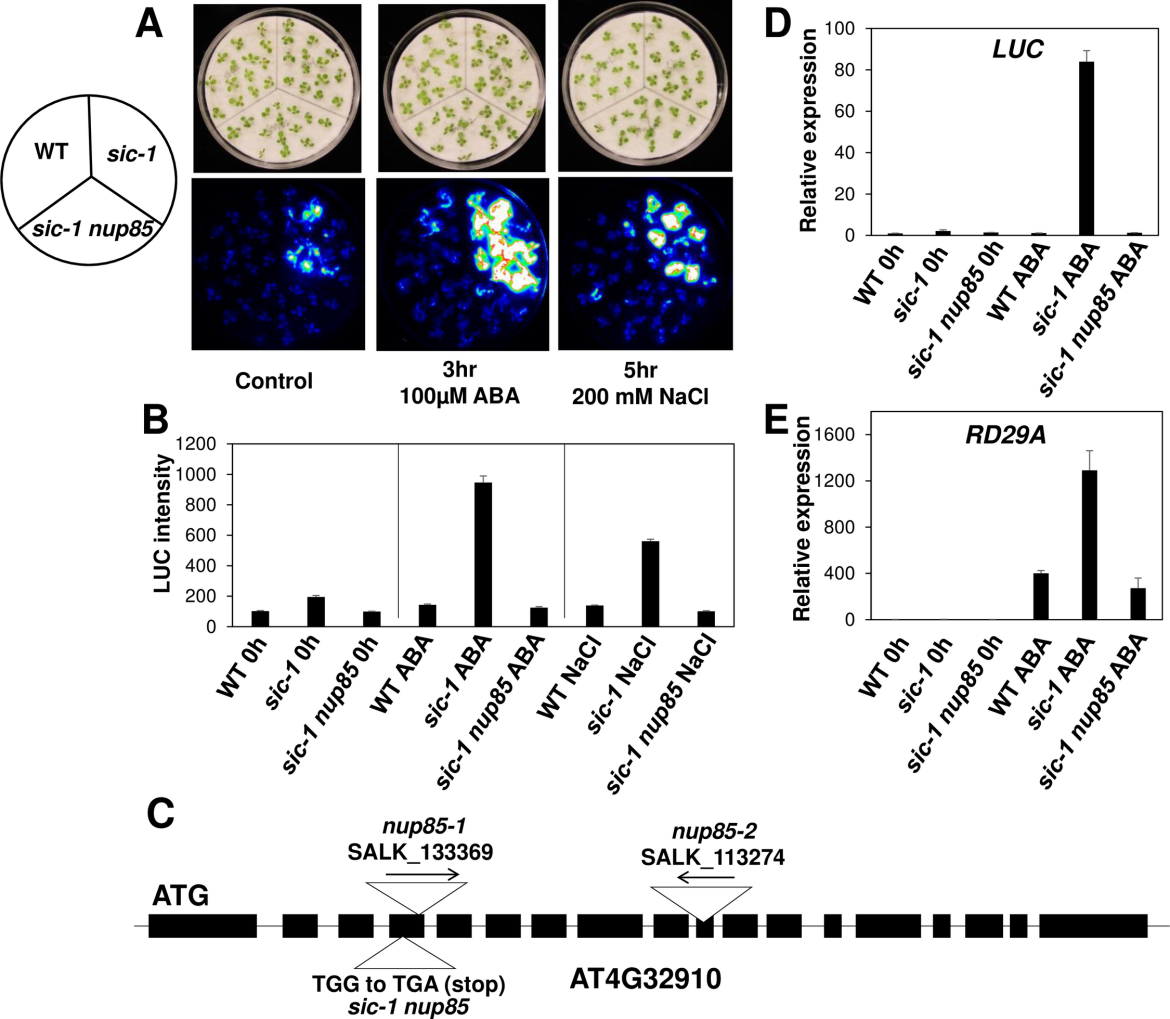
Isolation of *NUP85* as a suppressor of *sic-1* in response to ABA and salt stress. (A) Luminescence images of wild type, *sic-1* and *sic-1 nup85* double mutant seedlings under control, ABA and high salt treatments. (B) Quantification of the luminescence intensities of Fig 1A. (C) Diagram of *NUP85* genomic sequence and the three *nup85* mutants used in this study. The *sic-1 nup85* double mutant was caused by a G to A substitution which led to a pre-mature stop codon. (D) The expression of luciferase (*LUC*) in WT, *sic-1* and *sic-1 nup85* seedlings under control and ABA treatment. (E) The expression of *RD29A* in WT, *sic-1* and *sic-1 nup85* seedlings under control and ABA treatment. Total RNA was extracted from 7-day-old seedlings with mock or 50 μM ABA treatment. The relative transcript levels were normalized to *Arabidopsis Actin2* (*ACT2*) and the normalized expression level of WT at 0 h was set to 1. Data are present as mean value ± SD (n = 3).

To identify new factors involved in abiotic stress responses, we performed a forward genetics screen using an EMS mutant population generated in the *sic-1* mutant background with the stress-inducible *proRD29A-LUC* reporter gene. Putative mutants with altered luciferase (LUC) activities under abiotic stress treatments such as ABA treatment and salt stress were selected. One suppressor mutant was identified which exhibited reduced bioluminescence in response to ABA and salt stress compared to *sic-1* plants (Fig 1A). As revealed in Fig 1A and 1B, the luminescence representing *RD29A-LUC* expression was highly induced in *sic-1* by ABA and salt stress, whereas the luminescence was considerably weaker in the double mutant than *sic-1* after either ABA or salt stress treatment. The luminescence intensities of the suppressor mutant were similar to those in the Col *gl1* parental line harboring the *proRD29A-LUC* transgene (referred to as WT), which showed little LUC activity likely due to progressive transgene silencing [22,23]. After genetic mapping and whole genome re-sequencing, we discovered a mutation causing a premature stop codon in the 4^th^ exon in *NUP85* (AT4G32910), which encodes a nucleoporin protein (Fig 1C).

Gene expression data show that the *LUC* expression was highly induced in *sic-1* by ABA, but this induction was impaired in the suppressor (i.e. *sic-1 nup85*) mutant (Fig 1D). The transcript level of endogenous *RD29A* was induced by ABA treatment in WT, *sic-1* and the double mutant. However, the induction was significantly lower in the WT and *sic-1 nup85* double mutant compared to that in *sic-1* (Fig 1E). We also tested the expressions of other stress-responsive genes and the results showed that ABA-induced expression of *COLD-REGULATED 15A* (*COR15A*) and *RD29B* was lower in *sic-1 nup85* double mutants than that in *sic-1* mutant plants (S1 Fig).

**Fig 2:**
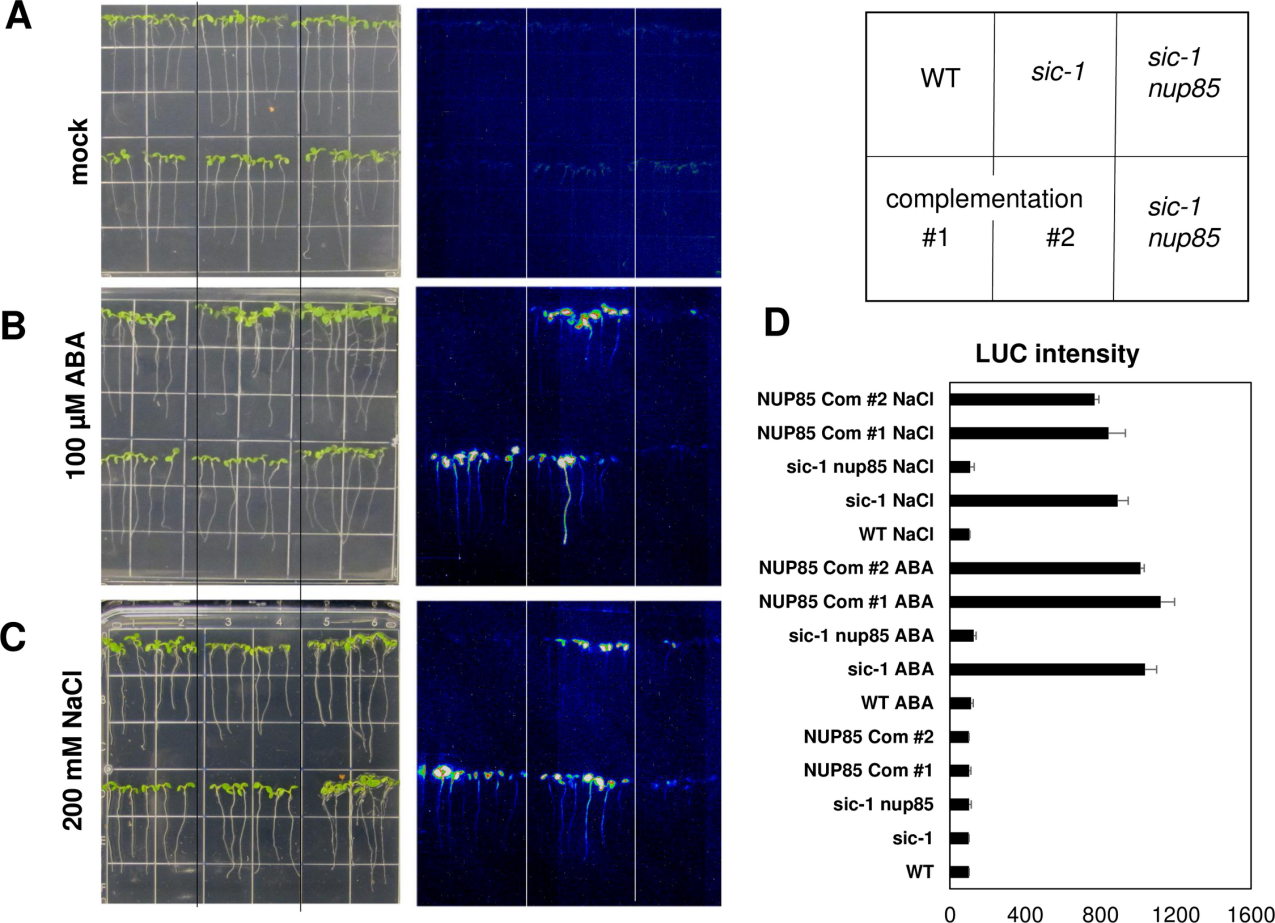
The reduced expression of *RD29A-LUC* in response to ABA and salt stress was rescued in *NUP85* complementation lines. The Luminescence images of WT, *sic-1*, *sic-1 nup85* double mutant and two independent NUP85 complementation lines with mock treatment (A), 200 mM NaCl treatment (B) and 100 μM ABA treatment (C). At least 20 seedlings of each genotype were treated with 100 μM ABA or 200 mM NaCl. Quantification of the luminescence intensities in A to C is present in (D). Error bars indicate SD. The experiments were conducted three independent times with similar results.

To verify if the suppressor phenotype was caused by the *NUP85* mutation, we cloned the genomic sequence of *NUP85* with its native promoter and generated two independent complementation lines by transforming *sic-1 nup85* double mutants. As shown in S2 Fig, the leaves of *sic-1 nup85* double mutant were bigger than *sic-1*. The wild type *NUP85* gene rescued the bigger leave size phenotype of the double mutant. We also examined the expression of *RD29A-LUC* reporter gene in the complementation lines after ABA and NaCl treatments (Fig 2). The diminished luminescence in *sic-1 nup85* double mutant was rescued in the *NUP85* complementation lines, which exhibited comparable LUC activity to *sic-1*. These genetic evidences demonstrated that the *NUP85* mutation is responsible for the double mutant phenotypes.

### The attenuated expression of stress responsive genes caused by *NUP85* mutation

**Fig 3:**
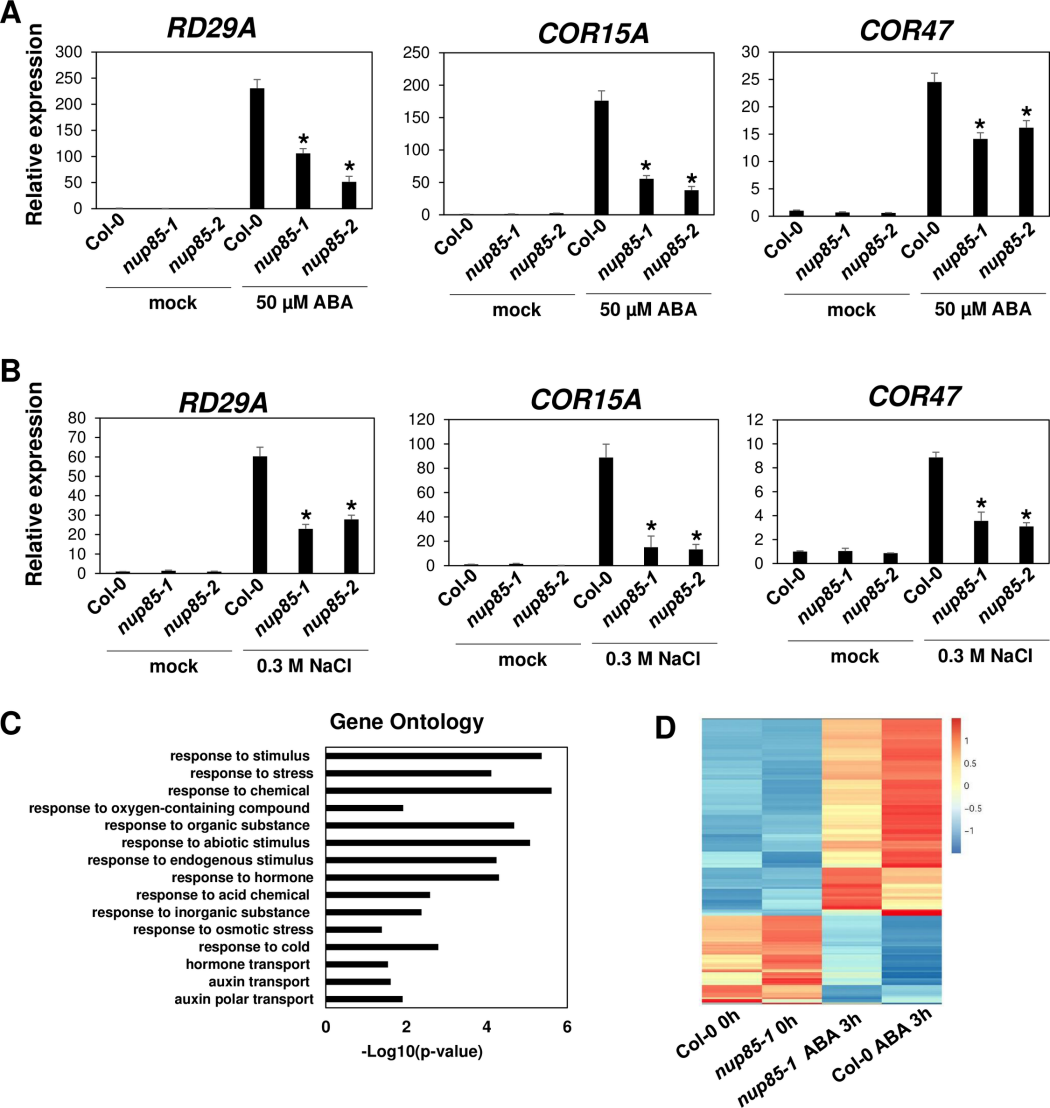
The attenuated expression of stress responsive genes in *nup85* mutants in response to ABA and salt stress. (A) The expression of stress responsive genes *RD29A*, *COR15A* and *COR47* in Col-0 wild type and two alleles of *nup85* mutants under mock and 50 μM ABA treatments for 3 h. The RNA was extracted from 7-day-old seedlings which were treated with mock or 50 μM ABA for 3 h. Data are mean values ±SD of three independent replicates. Asterisks indicate significant differences compared to WT Col under the same treatments. (B) The expression of *RD29A*, *COR15A* and *COR47* in Col-0 wild type and two mutant alleles of *nup85*. The RNA was extracted from 7-day-old seedlings which were treated with mock or 0.3 M NaCl for 5 h. Data are mean values ±SD of three independent replicates. Significance between mean values were analyzed by student’s *t* test (* P< 0.05). Asterisks indicate significant differences compared to WT Col under the same treatments. (C) Gene ontology enrichment analysis of the differentially expressed (DE) genes regulated by *NUP85* under mock condition (p value < 0.05). (D) Heat map depiction of the *NUP85* regulated ABA-responsive genes in Col-0 wild type and *nup85-1* mutants under mock and ABA treatments.

To investigate if *NUP85* affects the expression of endogenous stress responsive genes in response to ABA and salt stress, we obtained two independent T-DNA insertion homozygous mutants, *nup85-1* (SALK_133369) and *nup85-2* (SALK_113274) (Fig 1C). PCR analysis confirmed that the two T-DNA insertion mutant lines are homozygous (S3A Fig). Gene expression data further showed that *NUP85* expression was significantly lower in those two mutant lines compared to the Col-0 wild type (S3B Fig). We then treated the Col-0 wild type and two *nup85* mutants with 50 μM ABA for 3h, and found that the expression of several stress responsive genes such as *RD29A*, *COR15A* and *COR47* was significantly lower in the *nup85* mutants than that in the wild type after ABA treatment (Fig 3A). In addition to ABA, we also investigated if high salinity induced expression of stress responsive genes was also altered by the *NUP85* mutation. As shown in Fig 3B, salt stress induced expression of *RD29A*, *COR15A* and *COR47* was evidently impaired in the two *nup85* mutants after treated with 0.3M NaCl for 5h.

To better understand the genome-wide effects of *NUP85*, we performed RNA-sequencing experiments. Col-0 wild type and *nup85-1* mutant seedlings were treated with mock or 50 μM ABA for 3 hours. Under mock treatment, there were 197 differentially expressed (DE) genes which showed more than 1.5-fold changes in *nup85-1* mutant compared to Col-0 wild type plants (S1 Table). Gene ontology (GO) analysis revealed that the DE genes regulated by *NUP85* under mock conditions were enriched in categories such as responses to stimulus, response to stress and response to hormone, indicating an important role of *NUP85* in plant responses to environmental stresses (Fig 3C). Upon ABA treatment, there were totally 1389 ABA-responsive genes (759 ABA induced genes and 630 ABA repressed genes), whose expressions were significantly induced or repressed more than 4-fold changes by ABA treatments in Col-0 wild type (S2 Table), whereas there were 1357 ABA-responsive genes in *nup85-1* mutants after ABA treatments with 982 overlapping genes in Col-0 wild type (S3 Table). Out of 1389 ABA responsive genes in Col-0, 178 ABA responsive genes including *RD29B* exhibited significantly altered expressions in *nup85-1* mutants in comparison with Col-0 wild type, suggesting that about 13% of ABA responsive genes are regulated by *NUP85* (S4 Table). GO analysis further revealed that those *NUP85* regulated genes were also enriched at categories such as response to stimulus, response to chemical and response to abiotic stimulus (S4 Fig). It is possible that Nups may have redundant function in regulating ABA responsive genes since *nup85* single mutants did not alter most ABA-responsive genes. The heat map generated with *NUP85* regulated ABA- responsive genes in both Col-0 wild type and *nup85-1* mutant showed that the induction of a group of ABA-responsive genes was partially suppressed by the *NUP85* mutation (Fig 3D). In contrast, a small portion of DE genes were up-regulated in *nup85-1* mutant compared to Col-0 wild type. These results indicate that *NUP85* is involved in regulating gene expressions in response to ABA.

### The abiotic stress tolerance phenotypes of *nup85* and other nucleoporin mutants

**Fig 4:**
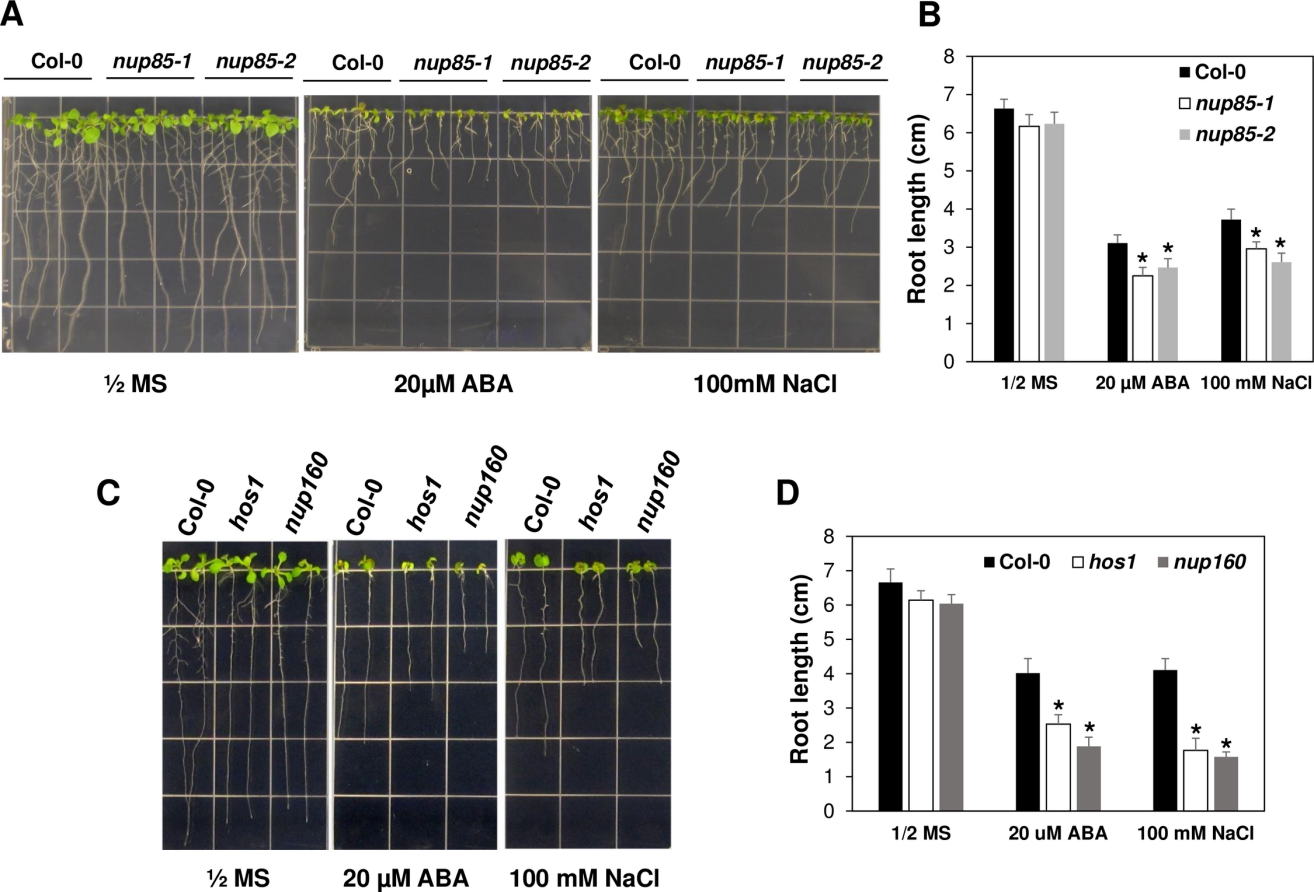
The hypersensitivity of *nup85*, *hos1* and *nup160* plants in response to ABA and salt stress. (A) The phenotypes of Col-0 wild type and two mutant alleles of *nup85* in response to ABA and NaCl. (B) Quantification of the primary root length of wild type, *nup85-1* and *nup85-2* at 7 days after transfer to the indicated media. (C) The ABA and NaCl inhibition of seedling growth of wild type, *hos1* and *nup160* mutants. (D) Quantification of the primary root length of wild type, *hos1* and *nup160* at 7 days after transfer to the indicated media. Phenotypes were documented at 7 days after the seedling transfer to ½ MS plates, 20 μM ABA or 100 mM NaCl containing medium. The experiments were repeated at least three independent times with consistent results. Values indicate means ± SD (n = 36). Significance between the mean values were analyzed with Student’s *t* test (* P< 0.05). Asterisks indicate significant differences compared to WT Col under the same treatments.

**Fig 5:**
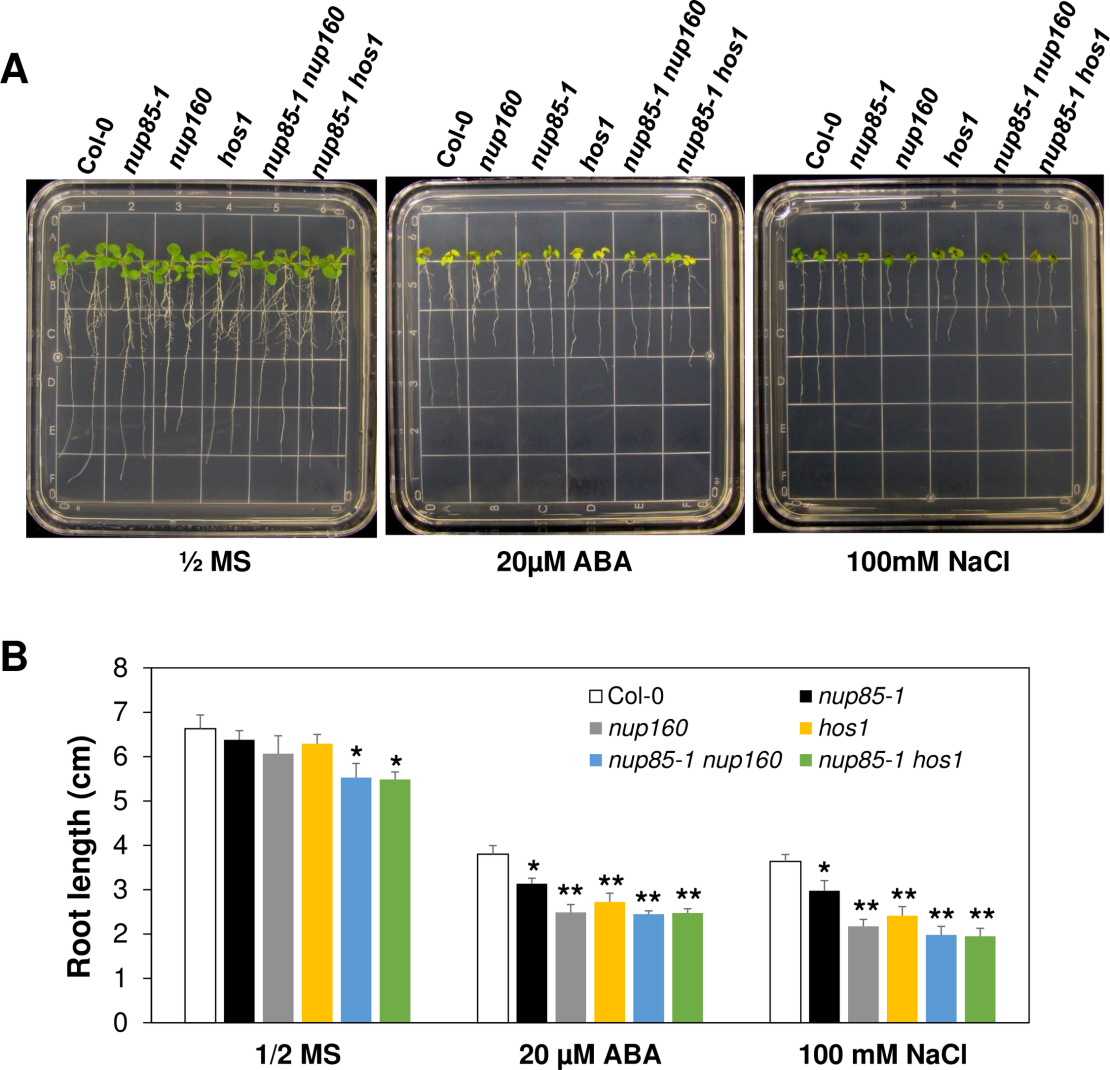
The ABA and salt hypersensitivity of *nup85-1 nup160* and *nup85-1 hos1* double mutants. (A)The root growth of Col-0 wild type, *nup85-1*, *nup160*, *hos1* single mutants and *nup85-1 nup160* as well as *nup85-1 hos1* double mutants on control ½ MS and ABA or NaCl-containing MS medium. (B)Quantification of the primary root length of indicated genotypes at 7 days after transfer to control and ABA or NaCl- containing MS medium. Three-day-old seedlings from each genotype were transferred to the indicated media and primary root length was measured after 7 days. Values indicate means ± SD (n = 24). Asterisks indicate significant differences compared to WT Col under the same treatments. Significance between the mean values were analyzed with Student’s *t* test (* P< 0.05, ** P< 0.01).

Since we have shown that *NUP85* is important for the expression of several stress responsive genes in response to ABA and salt stress, we next examined if *nup85* mutants may show altered stress phenotypes to ABA and salt stress. In plants, the NUP107–160 sub-complex consists of eight members, NUP160, NUP133, NUP107, NUP96, NUP85, NUP43, SEH1 and SEC13 [3,21,24]. Thus, we isolated homozygous mutant plants for the other seven nucleoporins and tested their stress phenotypes. As shown in S5 Fig, mutation of *NUP43*, *NUP107*, *NUP133*, *NUP96*, *SEC13* and *SEH1* did not cause obvious changes in their responses to ABA and salt stress when compared to Col-0 wild type control plants. The average root length of those mutants was similar to the Col-0 wild type control after being transferred to ABA and NaCl containing medium. Nevertheless, two alleles of *nup85* mutants were slightly more sensitive to ABA and salt stress as their root length was shorter than the wild type control after being transferred to ABA- and NaCl-containing medium (Fig 4A and 4B). In contrast, the root length of Col-0 wild type and *nup85* mutants was comparable when they were transferred to control medium. In addition to the *nup85* mutants, *hos1* and *nup160* mutants also showed increased sensitivity to ABA and salt stress as their root growth was significantly less compared to the Col-0 wild type after being transferred to the ABA and NaCl containing medium (Fig 4C and 4D). We also examined the responses of *nup85-1 nup160* and *nup85-1 hos1* double mutants to ABA and salt stress. As illustrated in Fig 5A, *nup85-1*, *nup160*, *hos1* single mutants and those two double mutants all displayed hypersensitivity to ABA and NaCl because their root length was shorter than Col-0 wild type after being transferred to the indicated ABA and NaCl containing medium. The root length of the double mutants was not evidently shorter than the single mutant (Fig 5B), indicating a lack of additive phenotypes in the double mutant, and thus suggesting that the Nups may function in the same genetic pathway in regulation of the tested stress responses. These genetic results suggest that *NUP85*, *NUP160* and *HOS1* are involved in ABA and salt stress responses.

### Expression of stress responsive genes is also attenuated in *nup160* and *hos1* mutants

**Fig 6:**
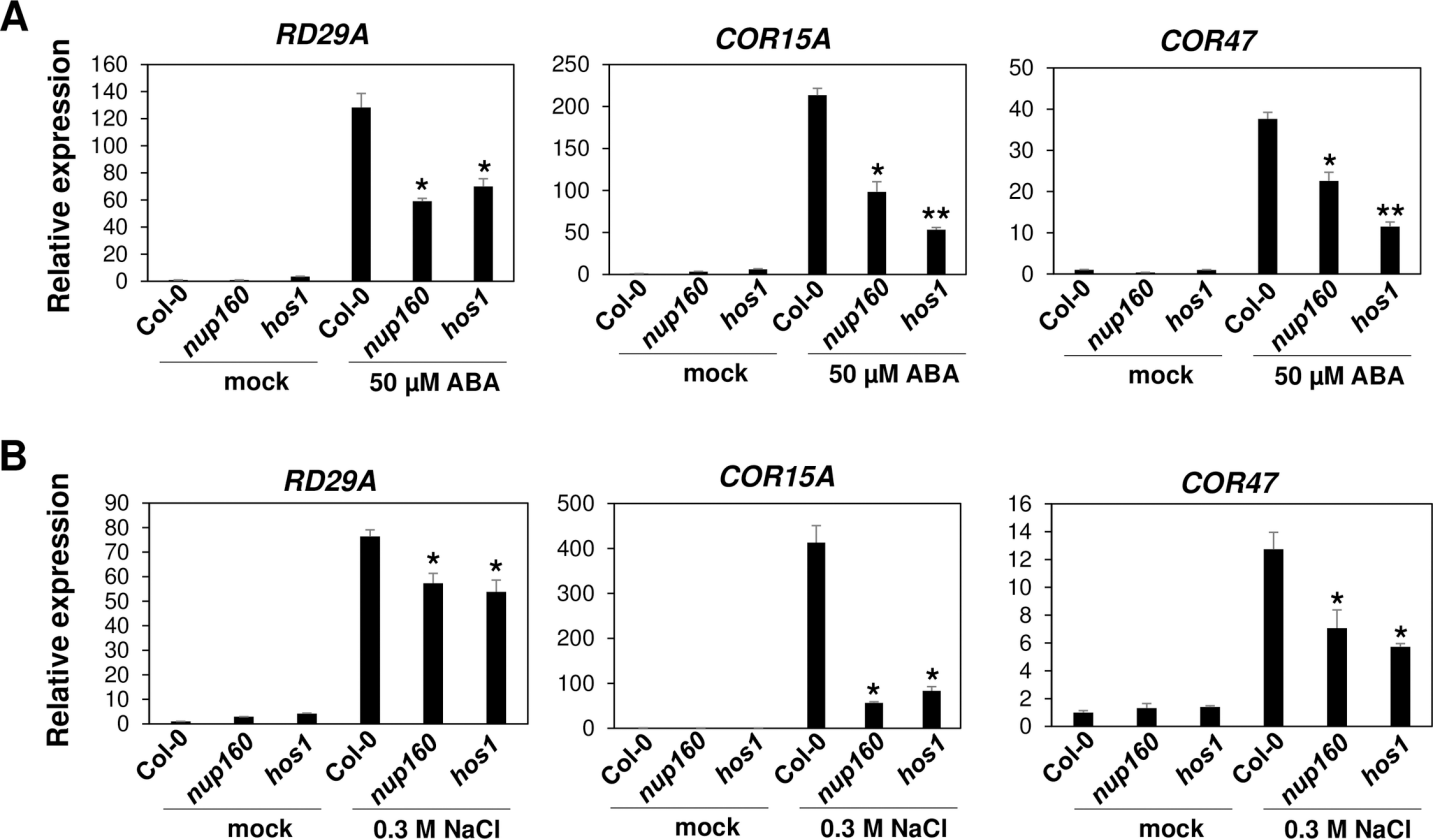
The impaired expression of stress responsive genes in *nup160* and *hos1* mutants in response to ABA and salt stress. (A)The expression of stress responsive genes *RD29A*, *COR15A* and *COR47* in Col-0 wild type, *nup160* and *hos1* mutants under mock and ABA treatment. (B)The expression of *RD29A*, *COR15A* and *COR47* in Col-0 wild type, *nup160* and *hos1* mutants under mock and salt treatments. RNA was extracted from 7-day-old seedlings which were treated with mock, 50 μM ABA or 0.3 M NaCl. Data are mean values ±SD of three independent replicates. Significance between mean values were analyzed by student’s *t* test (* P< 0.05, ** P< 0.01). Asterisks indicate significant differences compared to WT Col under the same treatments.

To investigate if *NUP160* and *HOS1* affect the expression of stress responsive genes in response to ABA and salt stress, we treated Col-0 wild type, *nup160* and *hos1* mutants with mock, 50 μM ABA or 0.3 M NaCl. The results showed that ABA and salt stress induced expression of *RD29A*, *COR15A* and *COR47* was expressively reduced in *nup160* and *hos1* mutants after ABA and salt treatment when compared to the Col-0 wild type (Fig 6A and 6B). Additionally, we also tested the expression of *RD29B* in the Col-0 wild type and those mutants. We found that the expression of *RD29B* was significantly inhibited in *nup160*, *hos1* and *nup85-1* mutants compared to Col-0 wild type (S6 Fig). These results suggest that *HOS1* and *NUP160* are also required for proper expression of stress responsive genes.

### Identification of putative NUP85 interacting proteins

Recent proteomics studies have revealed more than 22 Nups in plants [24]. To better understand the biological functions of NUP85 and its associating proteins, we performed anti-MYC immunoprecipitation and subsequent mass spectrometry (IP-MS) in WT and *Nup85pro*: *NUP85-MYC* transgenic plants. From two independent IP-MS experiments, we totally identified 55 putative NUP85 interacting proteins following two criteria to rule out the nonspecifically interacted proteins (1) proteins are present in the two independent replicates of IP-MS data; (2) proteins that have unique peptides or significantly more peptides (at least 5-fold more) identified in *NUP85* transgenic plants compared to WT samples. The full list of putative NUP85 interacting proteins are listed in S5 Table, NUP160, NUP133, NUP43, NUP96, NUP107 and Seh1 as well as Sec13 were present in the NUP85 immuno-complex with abundant unique peptides, confirming the conserved configuration of NUP107-160 sub-complex in plants. HOS1 was also present in the NUP85 immuno-complex with comparable abundance to other Nups in NUP107-160 complex. Besides Nups, some Transducin/WD40 repeat-like superfamily proteins such as Sec13A, which are known to be involved in mRNA and protein transport, were found in the NUP85 immuno-complex. Interestingly, several mediator subunits including MED16, MED14 and MED18 were also precipitated by NUP85, suggesting that possible involvement of NUP85 and other Nups in transcriptional regulation. To validate some of the candidate interacting proteins, we cloned HOS1 and Sec13A, and tested their interactions with NUP85 in split-luciferase (LUC) complementation assays. The results indicated that NUP85 could directly interact with Sec13A but not HOS1 (S7 Fig).

### The interaction between NUP85 and MED18

**Fig 7:**
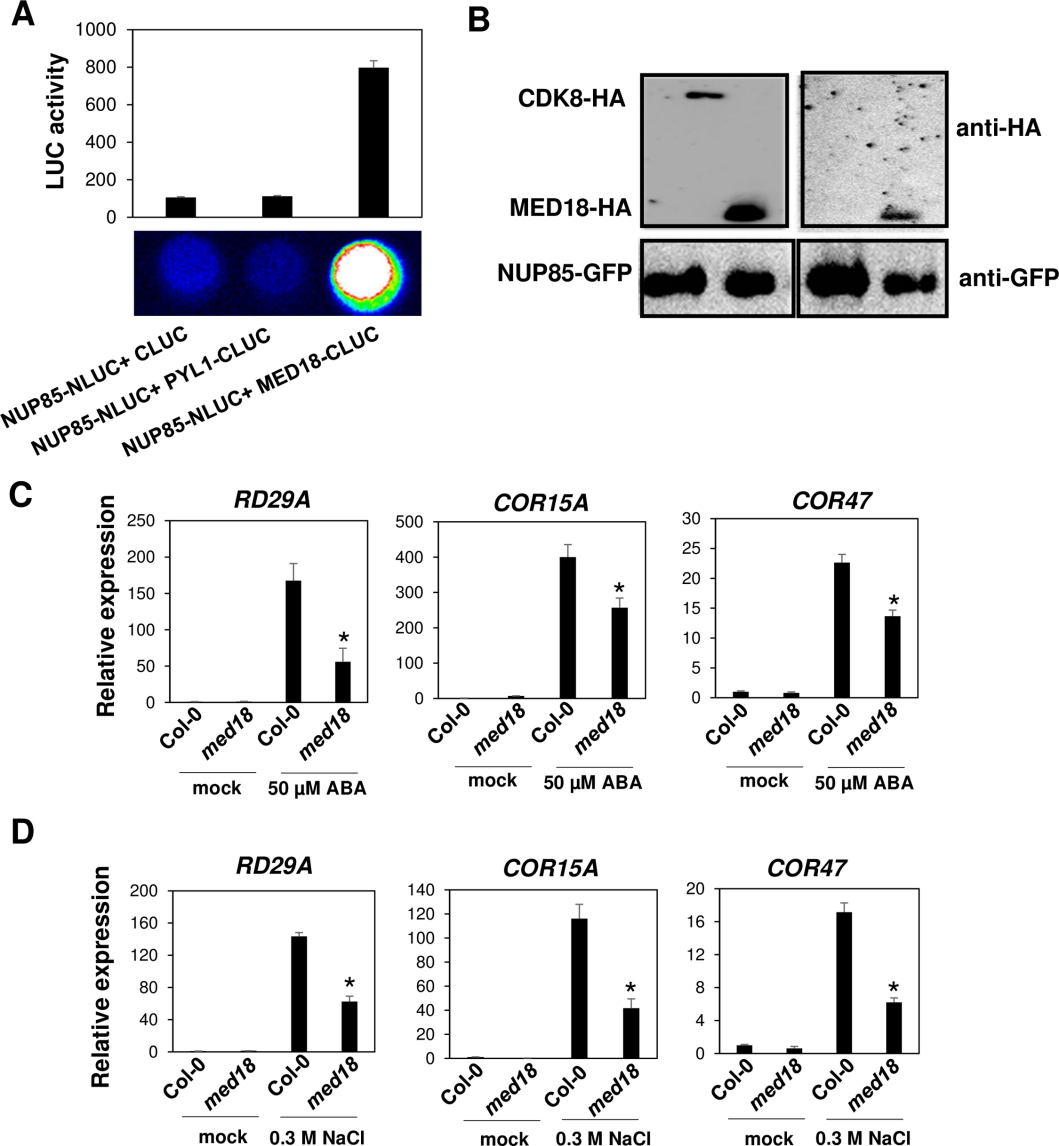
The interaction between NUP85 and MED18. (A) Split luciferase complementation assay in *Arabidopsis* protoplasts showing the interaction between NUP85 and MED18. Empty Cluc and PYL1-Cluc were used as negative controls. Approximately 1×10^4^ protoplasts per sample were co-transformed with indicated plasmids. The split-LUC complementation assay was repeated three independent times with consistent results. (B) Co-immunoprecipitation assays in *Arabidopsis* protoplasts confirming the interaction between NUP85 and MED18. CDK8-HA was used as a control. (C) The relative gene expression of *RD29A*, *COR15A* and *COR47* in wild type and *med18* mutants under mock or ABA treatments. (D)The relative gene expression of *RD29A*, *COR15A* and *COR47* in wild type and *med18* mutants under mock or salt treatments (0.3 M NaCl). In C and D, values indicate means ± SD (n = 3). Significance between the mean values were analyzed with Student’s *t* test (* P< 0.05).

To investigate if there is an interaction between NUP85 and mediator subunits, we selected MED18, which has been reported to be involved in ABA signaling pathway [25,26]. As shown in Fig 7A, the co-expression of MED18-Cluc and NUP85-Nluc resulted in strong LUC activity in the split-LUC complementation assay. In contrast, the co-expression of NUP85-Nluc and empty Cluc or PYL1-Cluc did not cause any detectable LUC activities. Co-immunoprecipitation assay in protoplasts showed that MED18-HA was precipitated by NUP85-GFP (Fig 7B), further confirming the interaction between NUP85 and MED18. In addition, we also investigated the temporal and spatial expression patterns of *NUP85* and *MED18* in Arabidopsis electronic fluorescent pictograph (eFP) browser. As illustrated in S8 Fig, both *NUP85* and *MED18* are widely expressed in most of tissues such as seeds, leaves, and stems, especially in shoot apex and flowers with higher expression levels.

**Fig 8:**
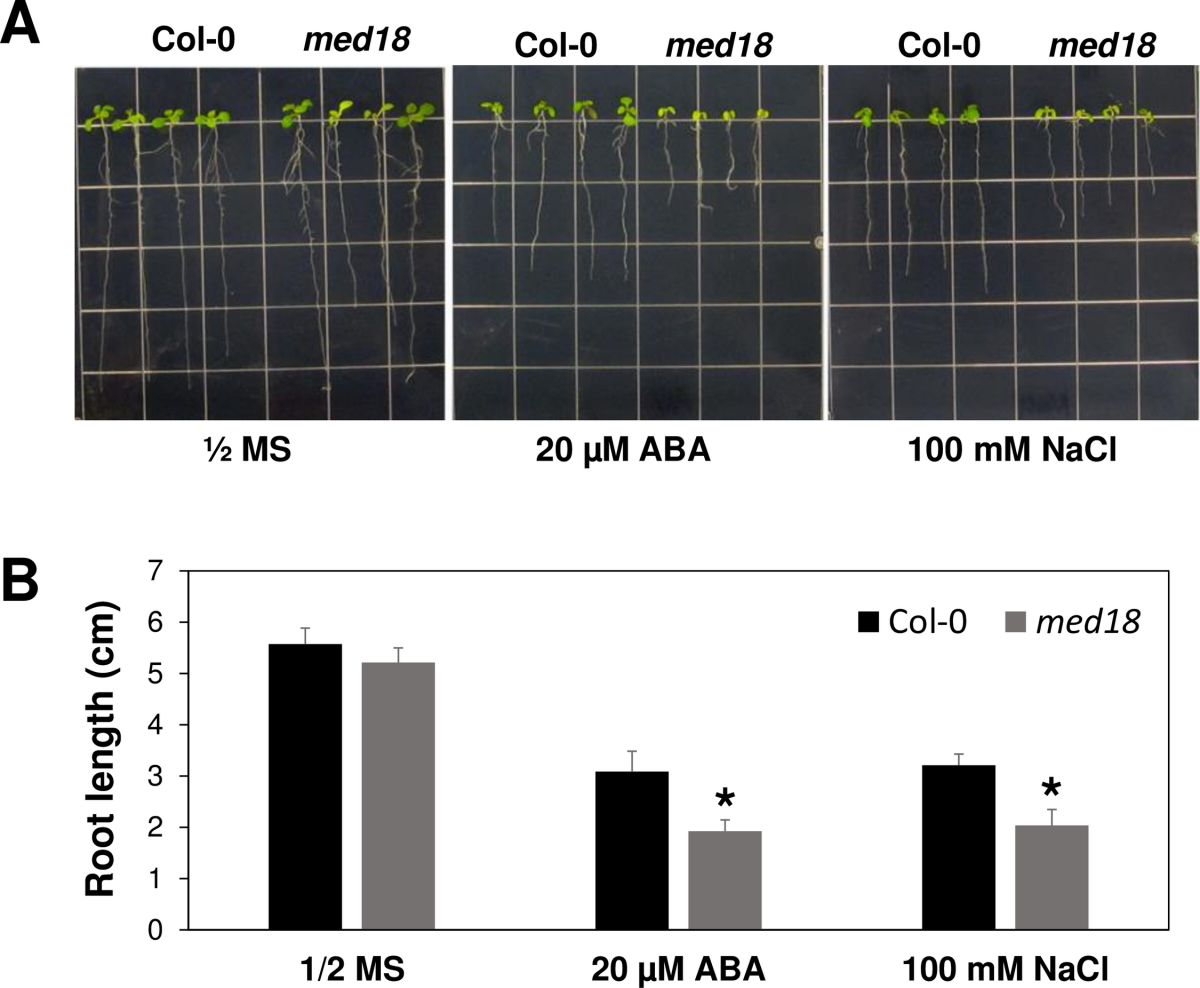
The hypersensitivity of *med18* in response to ABA and salt stress. (A) The root growth of Col-0 wild type and *med18* mutants on control ½ MS and ABA or NaCl-containing MS medium at 7days after transfer. (B) Quantification of the primary root length of Col-0 wild type and *med18* mutants at 7 days after transfer to control and ABA or NaCl- containing MS medium. Three-day-old seedlings were transferred to the ½ MS or ABA/NaCl containing media and primary root length was measured after 7 days. Values indicate means ± SD (n = 20). Asterisks indicate significant differences compared to WT Col under the same treatments. Significance between the mean values were analyzed with Student’s *t* test (* P< 0.05).

We next examined ABA and salt stress induced gene expression in Col-0 wild type and *med18* mutants (Fig 7C). Similar to *nup85* mutants, the ABA and salt stress induced expression of *RD29A*, *COR15A* and *COR47* was also inhibited in *med18* mutants relative to the wild type Col-0, signifying overlapping functions of MED18 and NUP85. To further explore the mechanism of how Nups regulate the expression of stress responsive genes, we examined *ABI5* expression in wild type Col-0, *nup85-1*, *nup160* and *hos1* as well as *med18* mutants under mock or ABA treatments. *ABI5* is not only one of the MED18 targeted genes [25], but also a key regulator for the expression of stress responsive genes [27]. As illustrated in S9 Fig, *ABI5* expression was obviously inhibited in those *Nup* mutants and *med18* mutants after ABA treatments when compared to the wild type Col-0. Consistent with the interaction between NUP85 and MED18, *med18* mutants also displayed hypersensitivities to ABA and salt stress because their primary root length was shorter than Col-0 wild type after transferred to MS medium containing indicated concentrations of ABA and NaCl (Fig 8).

## Discussion

Although core factors in abiotic stress signaling and responses have been identified with extensive genetic and biochemical studies in plants [28], abiotic stress responses remain intricate as many other factors yet to be discovered. In our present study, we have identified *NUP85* as a regulator of ABA and salt stress responsive genes. The mutation of *NUP85* impaired the ABA- and salt stress-induced expression of *RD29A* and several other stress-responsive genes, suggesting that *NUP85* may be a positive regulator for ABA and salt responses. Similarly, mutation of other Nups such as *HOS1* and *NUP160* also reduced the expression of stress responsive genes, thereby indicating that *NUP85* and other Nups components have overlapping functions in regulating the expression of stress responsive genes. Consistent with the altered expression of stress responsive genes, *nup85*, *nup160* and *hos1* mutants are hypersensitive to ABA and salt stress. Thus, our results indicate the involvement of Nups in ABA signaling and salt stress responses.

Currently, one of the best characterized NPC components is HOS1, which has been shown to associate with the chromatin of *FLOWERING LOCUS C* (*FLC*) and the promoter region of *MIR168b*, to regulate flowering time and miRNA biogenesis [15,23]. Nevertheless, how other Nups regulate gene expression remains largely unknown. Based on previous and our present studies, NPC components were suggested to play a role in gene expression regulation through various mechanisms. In fact, a number of previous studies have revealed that mutations in NPC components resulted in the accumulation of mRNAs in the nuclei [3,7,8,29,30], which could subsequently affect the expression of genes such as stress responsive genes in plants. Moreover, increasing evidence supported NPC as gene-gating organelles which could recruit actively transcribed genes to them and regulate gene expression [31]. It is likely that transcription of group of stress responsive genes is dependent on certain NPC components in response to different environmental stresses. Our results showed that the expression of *RD29A* and several stress responsive genes was impaired at the transcriptional level in *nup85*, *hos1* and *nup160* mutants, indicating that these NPC components are required for high level transcription of the stress responsive genes in response to ABA and salt stress. Additionally, plant NPC was recently demonstrated to undergo conformational switch in response to pathogen infection, allowing significant activation of nucleocytoplasmic transport and multiple stress-related signaling pathways [32]. The study raised the possibility that NPC components may facilitate the transcription of stress responsive genes in response to various environmental stresses by controlling the transport of critical signaling components and transcriptional regulators between the nuclei and cytoplasm.

In addition to these evidence, proteomics data from our studies further revealed several putative NUP85 interactors, which not only broadened our understanding of NPC functions in plants, but also indicated some potential mechanisms of how NPC components regulate the gene expression. It is worth noting that all the proteins identified from our proteomics data are putative NUP85 interacting proteins until they are validated by further experiments. Our IP-MS results suggested that NUP85 forms a complex with HOS1 and seven other nucleoporins located in the NUP107-160 sub-complex in plants, thus confirming the conserved configuration of NUP107-160 sub-complex in eukaryotic cells. Additionally, some Transducin/WD40-repeat proteins were co-purified with NUP85 including Sec13A, which are involved in assembling NPC domains, signal transduction and mRNA and protein transport [24]. Another interesting discovery from our proteomics data was that several mediator subunits were also present in the NUP85 immuno-complex, providing an alternative way for NUP85 and other Nups to regulate transcription. Recent studies in yeast cells showed that nuclear pore-associated TREX-2 complex directly interacts with mediators to regulate gene expression through the RNA Pol II transcription machinery [33]. However, whether NPC could regulate transcription through the mediator complex remains unknown in plants. Our data suggested the possibility of NUP85 and other NPC components in accessing the core transcriptional machinery through modulating the mediator complex and RNA Pol II association. Among the mediator subunits co-purified by NUP85, MED18 has been known to be involved in ABA signaling, flowering control and plant defense [25,34]. The ABA and salt stress induced expression of several stress responsive genes is significantly attenuated in *med18* mutants, which was similarly observed in *nup85* mutants. Importantly, we further demonstrated that NUP85 and MED18 are both important for the expression of *ABI5*, which could activate the expression of downstream stress responsive genes [35]. In summary, our study illustrates that NUP85 and some Nups such as NUP160 and HOS1 regulate stress-responsive gene expression through cooperating with mediator complex. Thus, our findings provide new insights into the role of Nups in ABA signaling and salt responses.

## Materials and methods

### Plant materials, growth conditions and stress phenotypic analysis

To screen for new regulatory components involved in abiotic stresses, we first performed EMS mutagenesis on *sic–1* mutants expressing RD29Apro-LUC as described in [23]. The mutants with significant reduced luminescence in response to 100μM ABA or 200 mM NaCl were selected. The wild type (WT) for LUC assays refers to Col–0 ecotype with the *gl–1* mutation harboring the *proRD29A-LUC* transgene. For abiotic stress phenotypic analysis and gene expression, WT refers to Col-0. The T-DNA insertion mutants *nup85-1* (SALK_133369C), *nup85-2* (SALK_113274C), nup160 (SALK_126801C), *hos1* (SALK_069312C), *nup43* (SALK_095344C), *nup107* (SALK_057072), *nup96* (SALK_135920C), *nup133* (SALK_092608C), *sec13* (SALK_045825C), *seh1* (SALK_022717C) and *med18* (SALK_027178C) were in Col-0 background and were obtained from the Arabidopsis Information Resource Center (ABRC). *nup85-1 nup160* double mutant was described in [36]. *nup85-1 hos1* double mutant was generated by crossing the nup85-1 and hos1 single mutant. The seeds were surface sterilized and sown on half Murashige and Skoog (MS) medium containing 1% sucrose and 0.8% agar. After 2 days in 4°C, the plates were moved to growth chamber under photoperiod of 16 h light/8 h dark. Root growth inhibition assays were performed as described previously [37]. For post-germination root growth assays, 3 or 4-days-old seedlings were first germinated on vertical half MS medium and then were transferred to ABA-(20 μM) or NaCl (100 mM) containing medium and the primary root growth was documented and quantitatively measured at 7 days after transfer.

### Generation of transgenic plants

To generate *NUP85* complementation lines, the genomic sequence of *NUP85* with about 2 kb upstream sequence before its start codon was amplified by high-fidelity DNA polymerase (PrimeSTAR HS DNA Polymerase, Clontech). The PCR product was first inserted into pENTR vector (Invitrogen), and then was transferred to destination vector pGWB-16 (MYC tag) via LR reaction. After verified by sequencing, the construct was introduced into Agrobacterium GV3101 for plant transformation. All indicated constructs were transformed by floral dip method [38]. The homozygous *NUP85* complementation lines were used for affinity purification.

### Luciferase imaging

Twelve-day-old seedlings of wild-type, *sic–1* and *sic–1 nup85* were sprayed with distilled water or indicated concentrations of ABA and NaCl solutions for 3h and 5h, respectively. The LUC images were captured using a low-light video imaging system with WinView software.

### Map-based cloning and whole genome sequencing

Map-based cloning was performed as described [23]. The F2 mutants with suppressed LUC phenotype were selected by genotyping for *sic-1* mutant. The results indicated that the mutation was located in chromosome 4 between 12,980 Kb and 16,500 Kb. Whole genome sequencing was performed, and a mutation was found in At4g32910 locus.

### Quantitative real-time PCR

RNA was extracted from 7-day-old seedlings by RNeasy Plant mini kit (QIAGEN) followed by DNase (Turbo) digestion. For testing gene expressions, 1 μg total RNA was used for reverse transcription using M-MLV reverse transcriptase (Promega) according to the manufacturer's instructions. Real-time PCR was performed using iQ SYBR Green Supermix (Bio-Rad) on a CFX96 real-time PCR detection system (Bio-Rad). *Actin 2* (*ACT2*) was used as the internal reference for all reactions. The relative gene expression value was calculated as described previously [39]. All primers were listed in S6 Table.

### RNA-sequencing and data analysis

wild type and *nup85* mutant seeds were germinated on 1/2 MS medium in growth chamber at 24°C for 7 days and were treated with mock or 3 hr of 50 μM ABA at room temperature. The total RNA was isolated with Trizol reagent (Invitrogen) according to the manufacturer’s instruction and RNA-sequencing were carried out by the Core Facility of Genomics in Shanghai Plant Stress Biology Center, China. Quality control was checked using FastQC (www.bioinformatics.babraham.ac.uk/projects/fastqc). RNA-Seq reads were trimmed using the fastx_trimmer command FASTX-Toolkit (hannonlab.cshl.edu/fastx_toolkit/index.html) with parameter “-f 11 -l 80” before alignment. Trimmed reads were mapped to *Arabidopsis* reference genome (TAIR10) using TopHat2 using “—b2-very-sensitive” option [40]. Read count for each gene were obtained using featureCounts [41]. Differentially expressed (DE) genes were identified using DESeq2 [42]. *NUP85*-regulated genes were defined under the criteria: 1) genes were differentially expressed after ABA in Col-0 with 4-fold change; 2) gene expressions in *nup85* mutants were at least 1.5-fold higher or lower than those in wild type after ABA treatments. GO enrichment analysis were performed at http://geneontology.org/.

### Affinity purification and mass spectrometry

For affinity purification of NUP85 and its associated proteins, about two grams of 12-day-old *NUP85pro*: *NUP85-MYC* transgenic seedlings were collected and same amount of WT seedlings was used as a negative control. Total protein extraction and affinity purification were performed as described previously [39]. The protein supernatants were incubated with 30 μL of anti-MYC agarose beads (Abcam), which had been pre-equilibrated with lysis buffer. After incubation at 4°C with rotation for 4 hours, the agarose beads were washed four times with lysis buffer followed by one time wash with 1 mL of PBS buffer. The agarose beads were finally resuspended in 100 μL of PBS buffer. After trypsin digestion, the mass spectrometer was operated in the data-dependent mode in which a full MS scan (from m/z 350–1500 with the resolution of 30,000 at m/z 400), followed by the 10 most intense ions being subjected to collision-induced dissociation (CID) fragmentation. CID fragmentation was performed and acquired in the linear ion trap (normalized collision energy (NCE) 30%, AGC 3e4, max injection time 100 ms, isolation window 3 m/z, and dynamic exclusion 60 s) according to [43]. The raw files were searched directly against the *Arabidopsis thaliana* database (TAIR10) with no redundant entries using Proteome Discover 2.1. Precursor mass tolerance was set at 10 ppm, and the MS/MS tolerance was set at 0.6 Da. The searches were performed with trypsin digestion and allowed a maximum of two missed cleavages on the peptides analyzed from the sequence database. The false discovery rates for proteins and peptides were set at 0.01, and the minimum peptide length was six amino acids. To identify significantly changed proteins from IP-MS results, we performed two biological replicates. The putative interacting proteins of NUP85 were selected based on two criteria:(1) proteins were identified in both replicates of IP-MS data or (2) proteins that have unique peptides or significantly more peptides in *NUP85* transgenic plants than in control samples.

### Split Luciferase (LUC) complementation

The full-length coding sequences of *NUP85*, *HOS1*, *Sec13A* and *MED18* were amplified by PCR using primers listed in S2 Table. The PCR products were first cloned into pENTER vector (Invitrogen) and then transferred to nLUC/cLUC vectors via LR reactions. Split-LUC complementation assay was performed in *Arabidopsis* protoplasts per [39]. The reconstitute LUC activity was detected in the dark by cooling camera. The image and quantification of LUC activities were analyzed with Winview software.

### Co-Immunoprecipitation assay

The NUP85-GFP and MED18-HA or CDK8-HA were transiently co-expressed in *Arabidopsis* protoplasts per [39]. After transformation, the protoplasts were collected and suspended in 1 mL lysis buffer containing 50 mM Tris-HCl, pH 7.5, 150 mM NaCl, 5 mM EDTA, 1 mM phenylmethylsulfonyl fluoride (PMSF), 2 mM DTT, 0.1 (v/v) Triton X-100 and Protease Inhibitor Cocktail (Sigma-Aldrich) in ice for 20 min. The lysis was then centrifuge at 13000 rpm for 15 min at 4°C and was incubated with pre-equilibrant GFP-Trap beads (Chromo Tek) for at least 4 hours with continuous rotation. The beads were washed at least four times with lysis buffer at 4°C and boiled in 4× SDS loading buffer for 10 min. Protein samples were separated by SDS-PAGE and were further detected with polyclonal anti-HA (Abcam) and anti-GFP antibody (Roche).

## Supporting information

S1 Fig The expressions of *COR15A* and *RD29B* in WT, *sic-1* and *sic-1 nup85* double mutants under mock and ABA treatment. RT-qPCR analysis showed that the expressions of ABA responsive genes *COR15A* and *RD29B* were significantly lower in *sic-1 nup85* double mutants when compared to *sic-1*. Values represent means ± SD (n = 3).
(TIF)

S2 Fig The leaf size and morphology of WT, *sic-1*, *sic-1 nup85* double mutants and *NUP85* complementation lines. (A) The typical leaves detached from indicated 5-week-old plants grown in soil. (B) The morphology of indicated genotypes grown in soil under normal growth conditions.
(TIF)

S3 Fig Verification of *nup85* T-DNA insertion mutants. (A) genomic DNA PCR showing the homozygous of *nup85* mutants. (B) The relative gene expression levels of *NUP85* in Col-0 wild type and two lines of *nup85* mutants. The RNA was extracted from leaves of 4-week-old plants in soil. Data represent means value ± SD (n = 3). Significance between mean values were analyzed by student’s *t* test (* P< 0.05). Significance between mean values were analyzed by student’s *t* test (* P< 0.05). Asterisks indicate significant differences compared to WT Col under the same treatments.
(TIF)

S4 Fig Gene ontology enrichment analysis of *NUP85* regulated ABA responsive genes (p value < 0.05). (TIF)

S5 Fig The root length of Col-0 wild type and nucleoporin mutants under control, ABA and salt stress. The root length was documented at 7 days after seedlings transfer to ½ MS plates, 20 μM ABA or 100 mM NaCl MS plates At least twelve 3-day-old seedlings from each genotype were transferred and root length was measured after 7 days. The experiments were repeated two independent times. Values indicate means ± SD (n = 24).
(TIF)

S6 Fig The expression of *RD29B* in Col-0 wild type, *hos1*, *nup160* and *nup85-1* mutants under mock and ABA treatments. Values represent means ± SD (n = 3). Asterisks indicate significant differences compared to WT Col under the same treatments. Significance between mean values were analyzed by student’s *t* test (* P< 0.05).
(TIF)

S7 Fig NUP85 interacts with Sec13A but not HOS1. Split-LUC complementation assays showing the interactions between NUP85 and Sec13A or HOS1 in *Arabidopsis* protoplasts. Approximately 1×10^4^ protoplasts per sample were co-transformed with indicated plasmids. The split-LUC complementation assay was repeated three independent times with similar results.
(TIF)

S8 Fig The expression patterns of *NUP85* and *MED18* in Arabidopsis eFP Browser. The snapshot of tissue specific expression patterns of *MED18* and *NUP85* from Arabidopsis eFP Browser.
(TIF)

S9 Fig The relative expression of *ABI5* in wild type, *nup85-1*, *nup160* and *hos1* under mock or ABA treatments. Values indicate means ± SD (n = 3). Asterisks indicate significant differences compared to WT Col under the same treatments. Significance between the mean values were analyzed with Student’s *t* test (* P< 0.05).
(TIF)

S1 Table The list of differentially expressed genes regulated by NUP85 under mock conditions. (XLSX)

S2 Table The list of ABA responsive genes identified in Col-0 wild type. (XLSX)

S3 Table The list of ABA responsive genes identified in *nup85-1* mutants. (XLSX)

S4 Table The list of ABA responsive genes regulated by *NUP85* in response to ABA. (XLSX)

S5 Table The list of putative NUP85 interacting proteins identified from IP-MS experiments. (XLSX)

S6 Table The list of primers used in the study. (XLSX)
