# Supplementary figures and images for: An Arabidopsis Nucleoporin NUP85 modulates plant responses to ABA and salt stress
Source: PLoS Genet. 2017 Dec 12;13(12):e1007124. doi: 10.1371/journal.pgen.1007124 (PMC5741264; doi:10.1371/journal.pgen.1007124)

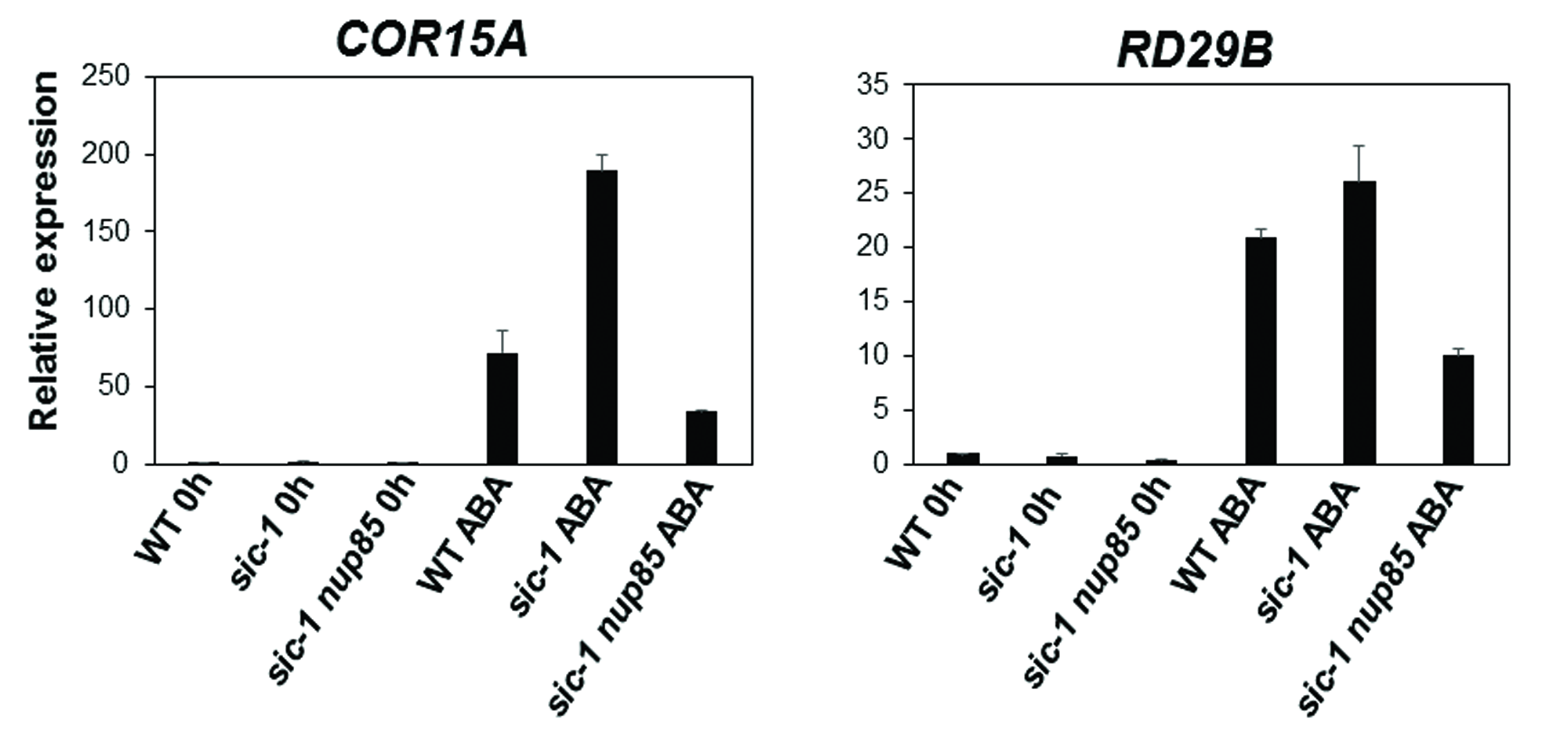

Supplement: S1 Fig — RT-qPCR analysis showed that the expressions of ABA responsive genes COR15A and RD29B were significantly lower in sic-1 nup85 double mutants when compared to sic-1. Values represent means ± SD (n = 3). (TIF) [file pgen.1007124.s001.tif]

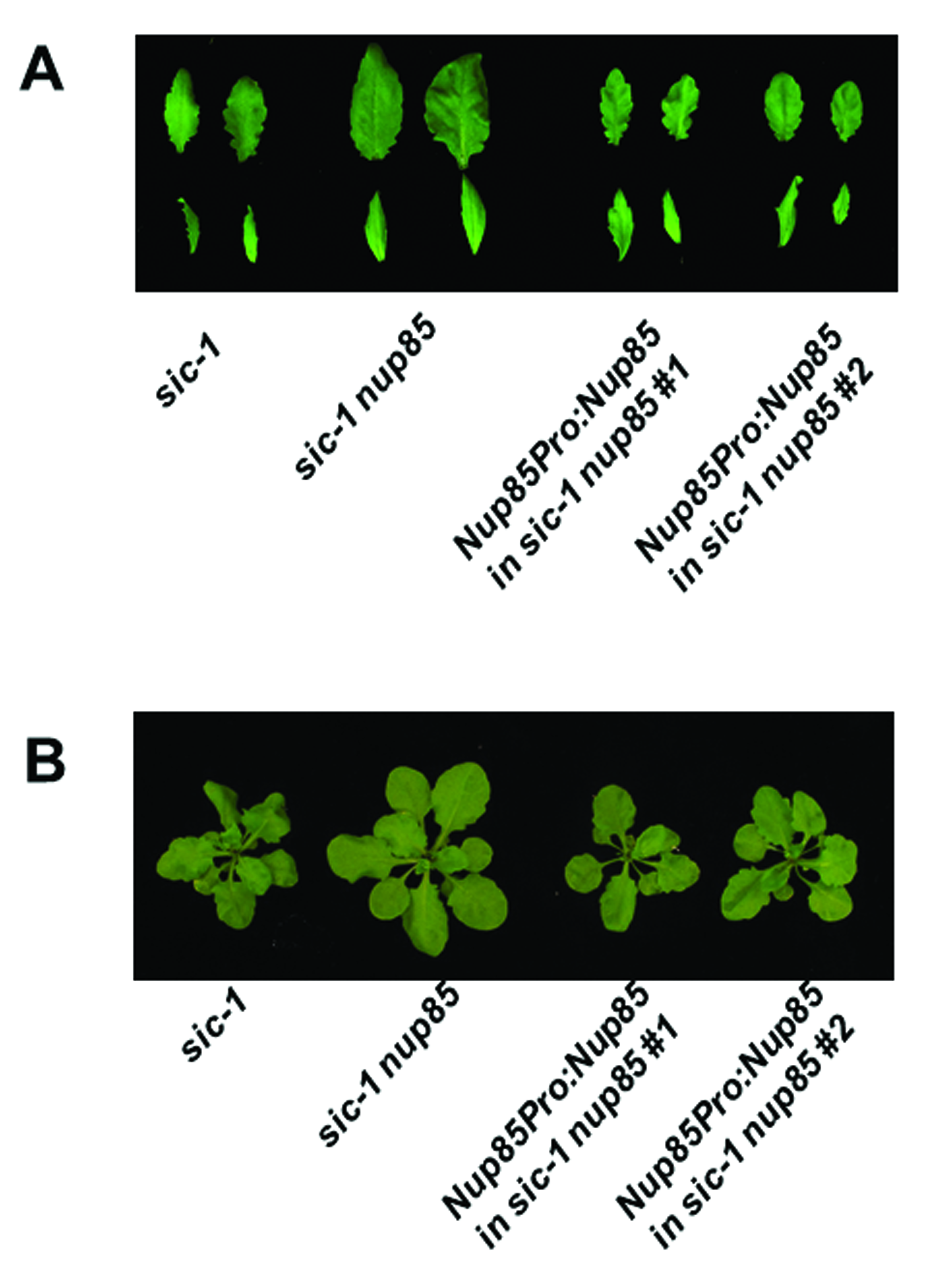

Supplement: S2 Fig — (A) The typical leaves detached from indicated 5-week-old plants grown in soil. (B) The morphology of indicated genotypes grown in soil under normal growth conditions. (TIF) [file pgen.1007124.s002.tif]

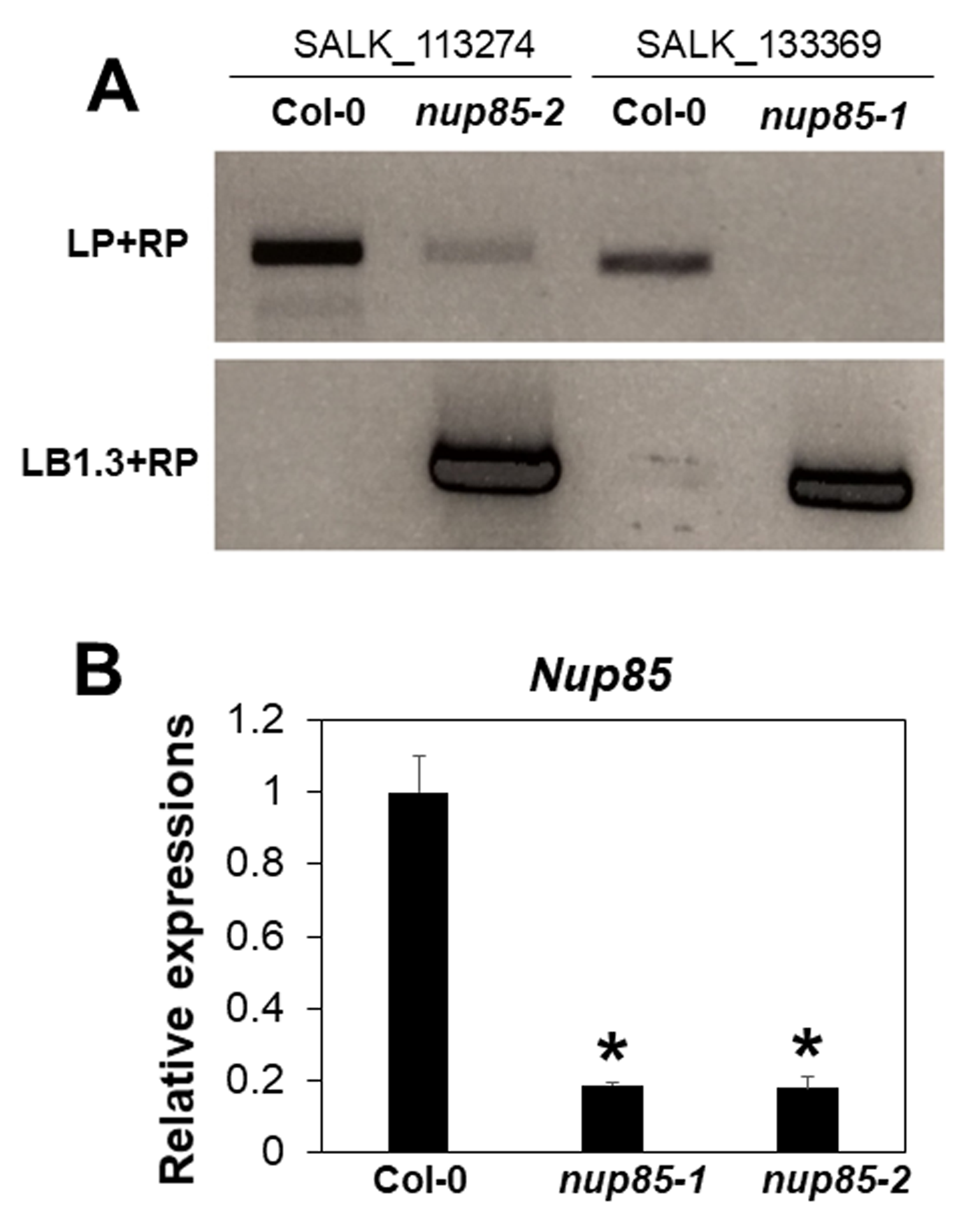

Supplement: S3 Fig — (A) genomic DNA PCR showing the homozygous of nup85 mutants. (B) The relative gene expression levels of NUP85 in Col-0 wild type and two lines of nup85 mutants. The RNA was extracted from leaves of 4-week-old plants in soil. Data represent means value ± SD (n = 3). Significance between mean values were analyzed by student’s t test (* P< 0.05). Significance between mean values were analyzed by student’s t test (* P< 0.05). Asterisks indicate significant differences compared to WT Col under the same treatments. (TIF) [file pgen.1007124.s003.tif]

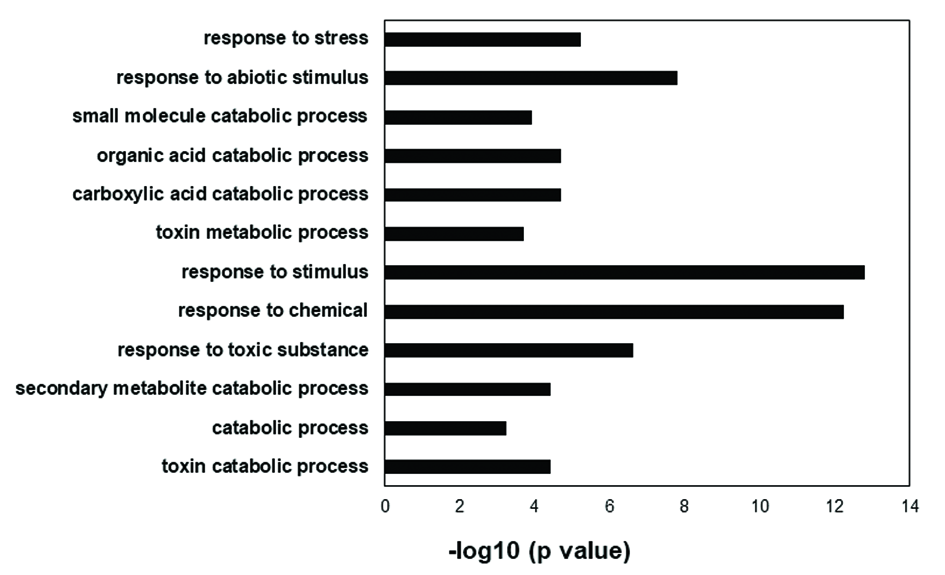

Supplement: S4 Fig — (TIF) [file pgen.1007124.s004.tif]

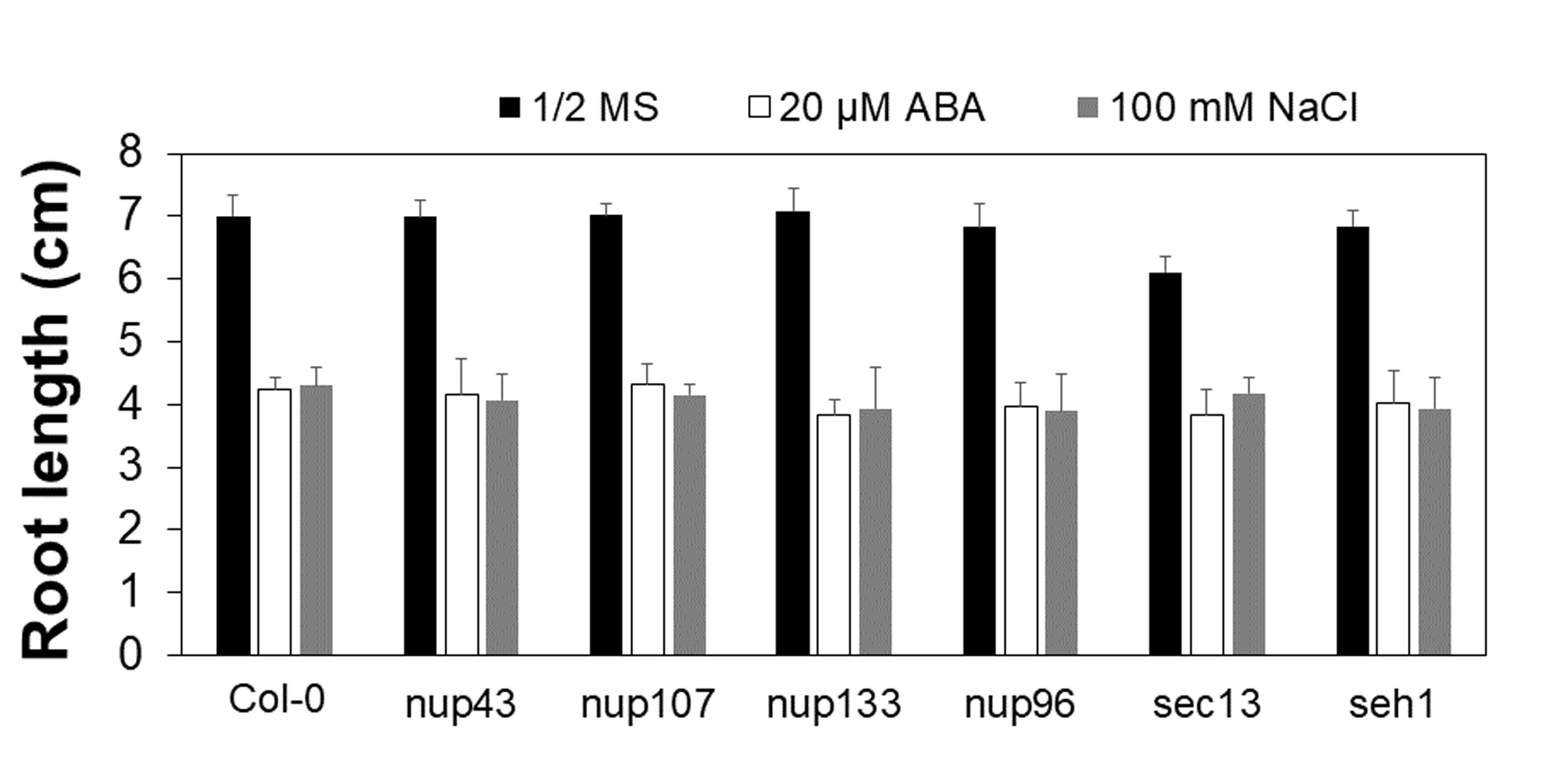

Supplement: S5 Fig — The root length was documented at 7 days after seedlings transfer to ½ MS plates, 20 μM ABA or 100 mM NaCl MS plates At least twelve 3-day-old seedlings from each genotype were transferred and root length was measured after 7 days. The experiments were repeated two independent times. Values indicate means ± SD (n = 24). (TIF) [file pgen.1007124.s005.tif]

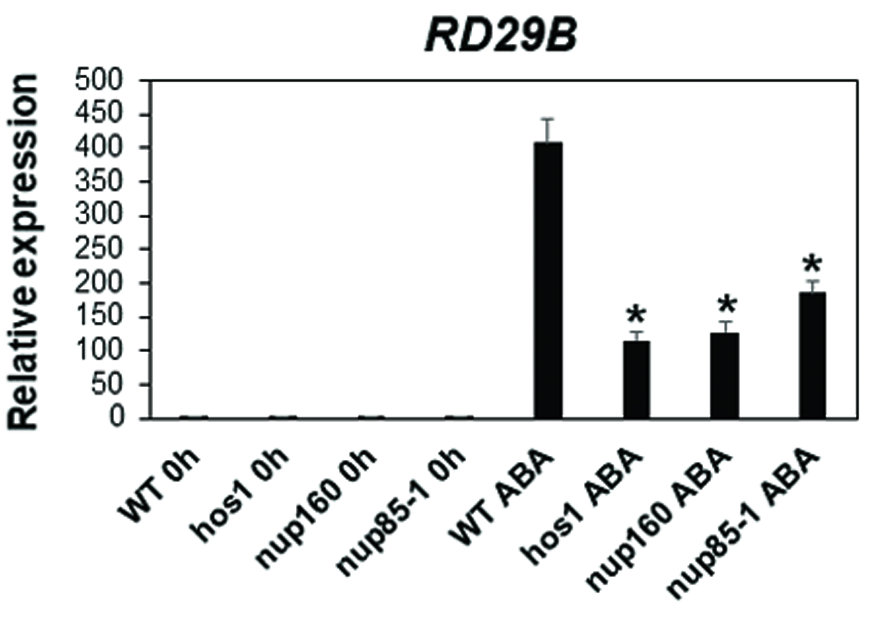

Supplement: S6 Fig — Values represent means ± SD (n = 3). Asterisks indicate significant differences compared to WT Col under the same treatments. Significance between mean values were analyzed by student’s t test (* P< 0.05). (TIF) [file pgen.1007124.s006.tif]

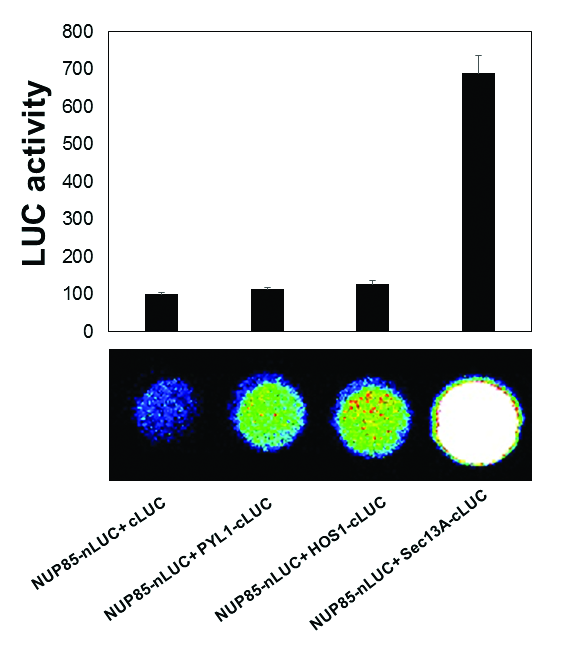

Supplement: S7 Fig — Split-LUC complementation assays showing the interactions between NUP85 and Sec13A or HOS1 in Arabidopsis protoplasts. Approximately 1×10^4^ protoplasts per sample were co-transformed with indicated plasmids. The split-LUC complementation assay was repeated three independent times with similar results. (TIF) [file pgen.1007124.s007.tif]

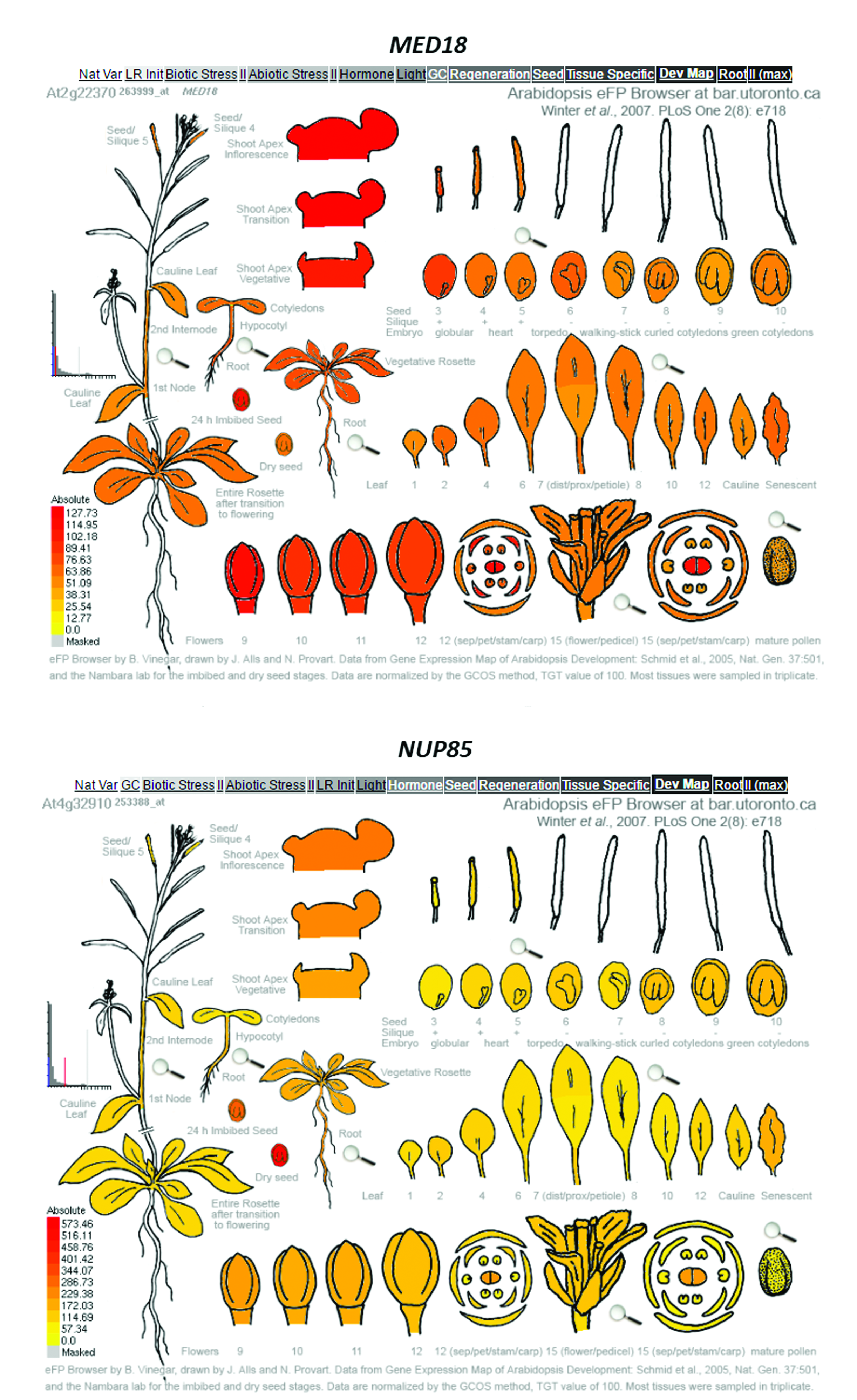

Supplement: S8 Fig — The snapshot of tissue specific expression patterns of MED18 and NUP85 from Arabidopsis eFP Browser. (TIF) [file pgen.1007124.s008.tif]

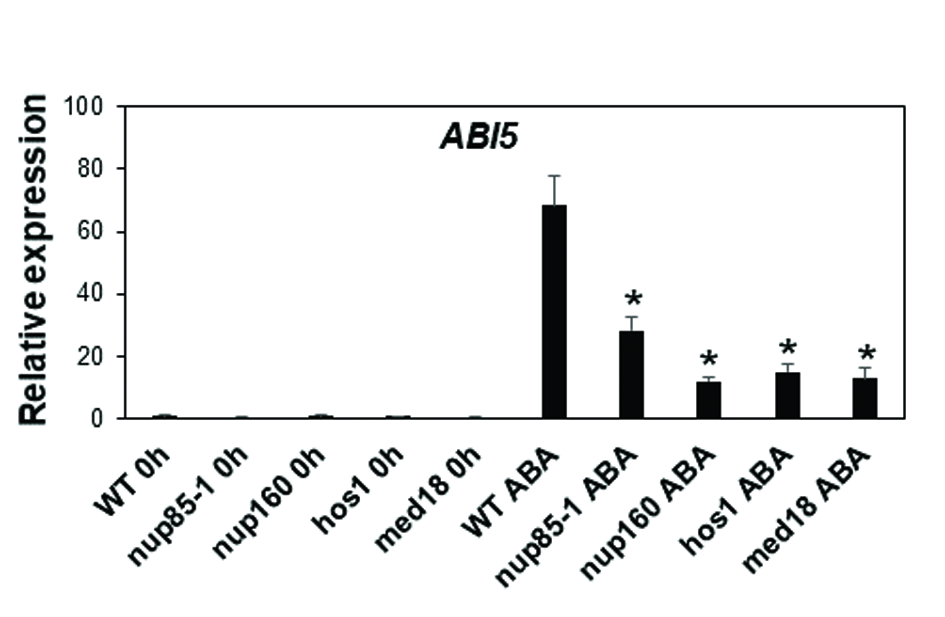

Supplement: S9 Fig — Values indicate means ± SD (n = 3). Asterisks indicate significant differences compared to WT Col under the same treatments. Significance between the mean values were analyzed with Student’s t test (* P< 0.05). (TIF) [file pgen.1007124.s009.tif]
